# Rumen fluke in Irish sheep: prevalence, risk factors and molecular identification of two paramphistome species

**DOI:** 10.1186/s12917-016-0770-0

**Published:** 2016-07-18

**Authors:** Ana Maria Martinez-Ibeas, Maria Pia Munita, Kim Lawlor, Mary Sekiya, Grace Mulcahy, Riona Sayers

**Affiliations:** Animal and Biosciences Department, Animal & Grassland Research and Innovation Centre, Teagasc, Moorepark, Fermoy, Co. Cork, Ireland; School of Veterinary Medicine UCD, Belfield, Dublin, Ireland

**Keywords:** *Calicophoron daubneyi*, *Paramphistomum leydeni*, PCR, Prevalence, Rumen fluke

## Abstract

**Background:**

Rumen flukes are trematode parasites found globally; in tropical and sub-tropical climates, infection can result in paramphistomosis, which can have a deleterious impact on livestock. In Europe, rumen fluke is not regarded as a clinically significant parasite, recently however, the prevalence of rumen fluke has sharply increased and several outbreaks of clinical paramphistomosis have been reported. Gaining a better understanding of rumen fluke transmission and identification of risk factors is crucial to improve the control of this parasitic disease. In this regard, a national prevalence study of rumen fluke infection and an investigation of associated risk factors were conducted in Irish sheep flocks between November 2014 and January 2015. In addition, a molecular identification of the rumen fluke species present in Ireland was carried out using an isolation method of individual eggs from faecal material coupled with a PCR. After the DNA extraction of 54 individual eggs, the nuclear fragment ITS-2 was amplified and sequenced using the same primers.

**Results:**

An apparent herd prevalence of 77.3 % was determined. Several risk factors were identified including type of pasture grazed, regional variation, and sharing of the paddocks with other livestock species. A novel relationship between the Suffolk breed and higher FEC was reported for the first time. The predominant rumen fluke species found was *C. daubneyi*. Nevertheless, *P. leydeni* was unexpectedly identified infecting sheep in Ireland for the first time.

**Conclusions:**

An exceptionally high prevalence of rumen fluke among Irish sheep flocks has been highlighted in this study and a more thorough investigation is necessary to analyse its economic impact. The isolation of individual eggs coupled with the PCR technique used here has proven a reliable tool for discrimination of *Paramphistomum* spp. This technique may facilitate forthcoming studies of the effects of paramphistomosis on livestock production. The most noteworthy finding was the identification of *P. leydeni* affecting sheep in Ireland, however further studies are required to clarify its implications. Also, a significant relationship between Suffolk breed and a heavier infection was found, which can be used as a starting point for future research on control strategies of rumen fluke infection.

## Background

Paramphistomes (rumen fluke) are cosmopolitan trematodes in ruminants and consist of a number of different species of the Paramphistomidae family. Like other trematodes, the life cycle of paramphistomes is indirect, and similar to *Fasciola hepatica*, involves a mollusc snail as an intermediate host. The parasite larvae develop in the snail until cercaria emerge and encyst on vegetation, hard surfaces, or in water. The encysted metacercaria are the infective stage which is ingested by ruminants, the final host. Juvenile paramphistomes first migrate to the small intestine and feed on the intestinal mucosa causing intestinal damage which, on occasion, is severe enough to result in death [1, 2]. Eventually, the flukes migrate upwards to the reticulum and rumen where they mature and produce eggs to complete the lifecycle.

Paramphistomosis is highly prevalent in tropical and subtropical countries [3–5] where it results in considerable morbidity. In Europe, however, it has been considered of minimal clinical significance for many years [6]. More recent studies have highlighted a sharp increase in the prevalence of rumen fluke infections in several European countries including Ireland [7–9]. Moreover, several reports of clinical paramphistomosis, with severe symptoms including death, have been described in both sheep and cattle [9, 10], and now it is being recognised as a clinical entity in Irish livestock. This parasitosis can therefore be considered an emerging parasitic disease in Europe [11], and hence, should be included in the differential diagnosis of enteric disease in all ruminants.

The detection of rumen fluke is most commonly carried out by conventional coprological techniques as a rapid immunological method is, as yet, unavailable. These techniques do not routinely allow the identification of the species of paramphistome based on the morphological characteristics of the eggs. There is also a lack of epidemiological information regarding paramphistomes in temperate regions, especially when compared with published data regarding *F. hepatica* (liver fluke). Controversy still exists over the taxonomic classification of paramphistomes and this is being reviewed across Europe [12]. Recent studies based on the specific identification of adult parasites collected in abattoirs suggest that the only species infecting domestic livestock is *Calicophoron daubneyi,* both in Europe [12–14], and more specifically in Ireland [15, 16]. Few studies have been carried out to determine the risk factors associated with the presence of rumen fluke in a sheep flock. Little data is also available with regard to the pathology of a rumen fluke infestation, and the economic impact of paramphistomosis remains equivocal [5].

Gaining a better understanding of rumen fluke transmission and identification of risk factors is critical to improve the control of rumen fluke infection. These studies are hindered by the lack of accurate techniques to identify the species of rumen fluke when using conventional coprological techniques. Differentiation of eggs at the species level is essential for epidemiological surveys, as different species of rumen fluke may have different distributions, host specificities and pathogenic outcomes. In this context, a number of molecular techniques have been developed to specifically identify several trematodes such as *Fasciola hepatica* or *Dicrocoelium dendriticum*, among others, although the number of studies in rumen fluke remains limited. The ITS2 (Internal transcribed spacer 2) region of the parasite genome is relatively conserved within a species or genus and has proven to be a useful marker in the discrimination of different parasite species including paramphistomes [17–20]. Amplification and sequencing of this region, therefore, provides a useful tool of identifying the species of rumen fluke present in a particular animal.

A previous Irish study [16], based on passive surveillance, highlighted a rise of rumen fluke infection across Irish livestock. The national prevalence of rumen fluke in Irish flocks, however, still remains unknown. Given the recent and on-going expansion of Irish livestock enterprises (Food Harvest 2020 document, 2010^1^), an increase in the prevalence of an emerging parasitic disease could represent a threat to farm profitability. With this in mind, prevalence data is crucial to design appropriate control strategies as is identification of the predominant species affecting sheep flocks. The aims of this current study, therefore, were (i) to document the prevalence of rumen fluke in Irish flocks using faecal examination, (ii) to identify risk factors associated with rumen fluke infestation in these flocks, and, (iii) to identify the species of paramphistome present in a selection of samples using egg isolation coupled to PCR amplification of the second internal transcribed spacer (ITS-2) region of the genome.

## Methods

### Sample population and submission of samples

The current study was conducted between November 2014 and January 2015, taking into account the risk period for fluke infection in this latitude. Farmer recruitment was carried out using Teagasc (Irish Food and Agriculture Development Authority) networks amongst Irish sheep farmers. This included direct recruitment by over 50 Teagasc sheep advisors based nationally, and advertising in two Teagasc publications which have a readership of approximately 40,000 farming clients (almost 50 % of the total farming population in Ireland). Applicants were subsequently stratified based on geographical location and flock size in order to more fully represent the Irish sheep flock population, those participants who didn’t fit the criteria were excluded from the study. Participation in the study was entirely voluntary and non-incentivised. A criterion for inclusion in the study was that the flock could not have been dosed with a flukicide within six weeks of sample collection.

A standardised sampling kit was posted to each farmer with a request to submit 20 fresh faecal catch samples from 20 different mature ewes. Each kit contained 20 sample pots, a pair of gloves, an instruction leaflet, a sample submission form, and a pre-paid, pre-addressed envelope for sample submission to the School of Veterinary Medicine laboratory, University College Dublin, Ireland, for faecal egg counting (FEC). Farmers were requested to post samples immediately after collection.

### Coprological technique

The 20 catch samples collected from ewes on each participating farm were combined into 2 pools of 10 samples for FEC analysis. The composite samples were prepared using 3 g of faeces from each sample pot, mixed well using a spatula, to yield two 30 g samples per farm. Subsequently, 5 g from each composite was homogenised with water and passed first through a coarse mesh sieve and then a finer 250 μm mesh sieve. The filtrate was allowed to stand for five minutes to sediment and the supernatant removed by aspiration. This sedimentation step was repeated up to two more times, if required. The supernatant was removed and sediment was stained with two drops of 1 % methylene blue. Eggs were counted on a stereomicroscope as outlined by [21]. Faecal egg counts were reported as eggs per gram of faeces (epg), assuming a test sensitivity of 90 %. The two results from each farm were averaged to yield a final FEC for each flock.

### Farm management data

A questionnaire survey was used to collect relevant farm management data, consisting on 21 semi-closed questions. The survey was divided into three sections; (i) farm and grazing management, which included predominant breed of sheep in each flock (e.g. Suffolk, Texel, Blackface Mountain), lambing period (early, mid, or late season), flock size, presence of other livestock on the farm e.g. cattle, type of grazing (lowland or mountain pastures), and sharing of paddocks by different livestock species; (ii) rumen fluke specific data which included, recording any occurrence of veterinary-diagnosed clinical cases of paramphistomosis, whether these cases included marked morbidity or death, and the frequency of clinical cases within the previous five years; and, (iii) chemoprophylactic treatments administered on the farm, including the type of product used, the times of year administered, and the date of administration of the most recently used anthelmintic. The survey was conducted as a postal survey.

### Flock classification

**Fig. 1:**
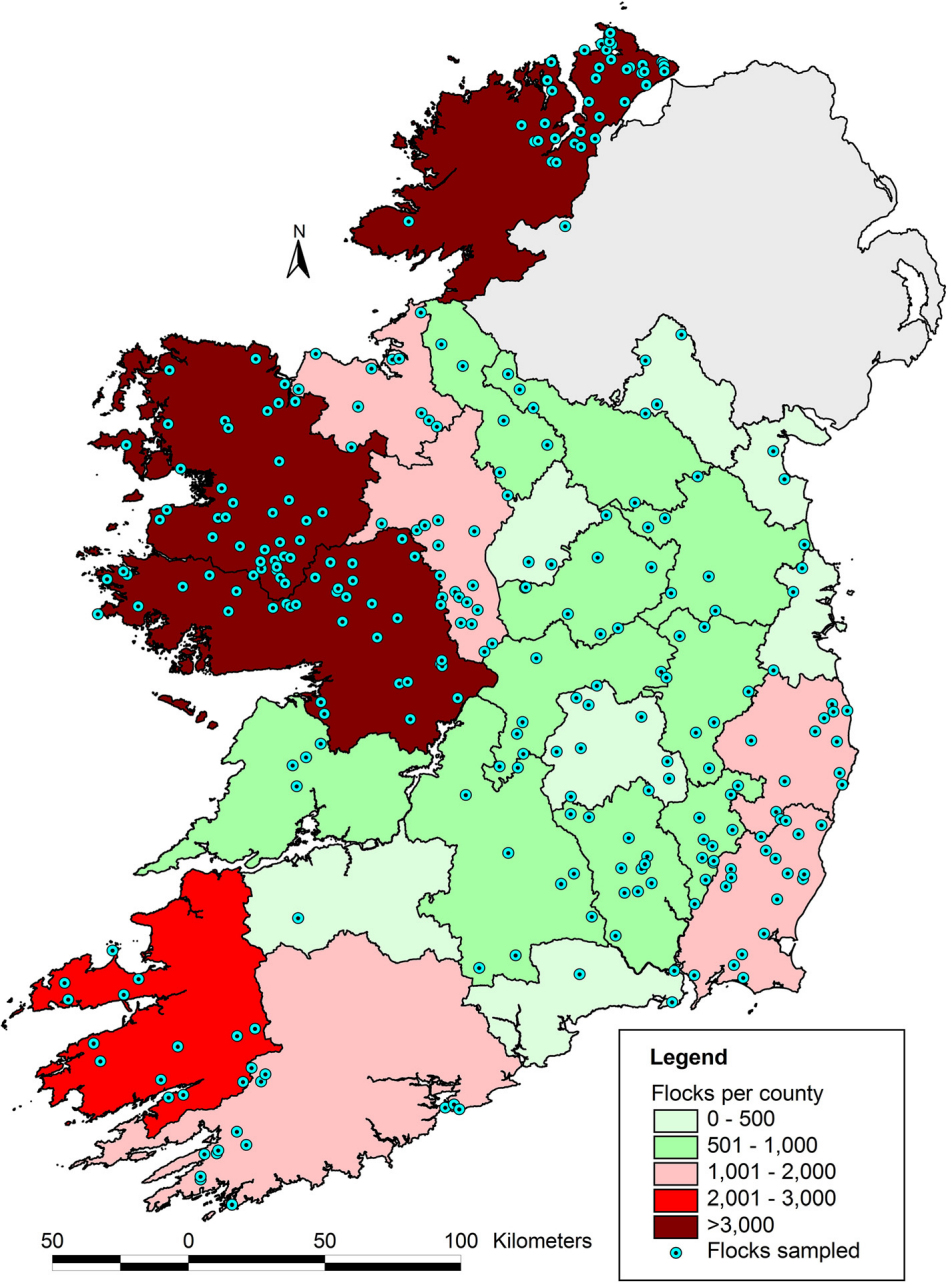
Map of Republic of Ireland outlining the location of study flocks within the counties

To compare the distribution of paramphistome burdens across different farms, flocks were classified into one of the following categories; free (0 epg), low (<20epg), medium (20–50 epg) and high (>50 epg faeces) [15]. Each flock was also categorised as positive or negative based on the presence of at least one egg in either of the composite samples. If no rumen fluke eggs were detected using this strategy, a high probability of disease freedom existed assuming a within-flock prevalence of 20 % and a test sensitivity of 70 %. Flocks were also allocated in two different geographical regions (region 1 and region 2) based on the number of flocks per county as recorded in the national agricultural census (CSO, 2014). Region 1 was classified as the most sheep dense region (>1500 flocks), with region 2 representing counties with lower sheep numbers (Fig. 1). With regard to predominant breed in each flock, four categorisations were used to classify flocks i.e. Suffolk, Texel, Mountain breeds (Cheviot and Blackface Mountain), and others. Finally, flocks were classified on the basis of lambing season (early [December and January]; mid [February and March]; late [April, May, June]), rumen fluke dosing regimen (dosed[D], undosed flocks [UD]), flock size (1–50 ewes, 51–200 ewes, >200 ewes), type of farming enterprise (sheep only, mixed livestock), organic farm (yes, no), type of pasture available (lowland, mountain), and whether different species were mixed in the same grazing areas (yes, no).

### Prevalence data analysis

Hard copy postal surveys were manually entered into a web-based survey tool^2^ and visually checked by two researchers for accurate data entry. Coded responses to each question were subsequently downloaded into MS Excel (MS Office version, 2010) which was used to collate the data, fix variables for directionality, and generate graphical representations. Apparent prevalence (Ap) was calculated based on percentage of flocks recording positive results against the total sample population tested in the study. A test sensitivity of 90 and specificity of 99.9 % was assumed. True prevalence (Tp) was calculated using the Rogan-Gladen estimator in survey toolbox version 1.04 (www.ausvet.com.au [22]). Prevalence data were calculated both on a national and per county basis. Normality of the data was assessed visually using ladder of powers histograms, with normality of residuals assessed using normal probability plots and kernel density estimate plots constructed in Stata version 11 (StataCorp, USA). Pearson’s chi-squared test, Fisher’s exact test, *t*-test, and regression analyses were carried out using Stata.

All regression models were constructed by first completing either a chi-squared (categorical variables) or *t*-test (continuous variables) univariable analysis examining all two way associations. Those variables recording *P* values of ≤0.15 in univariable analyses were included in multivariable models. A manual backwards elimination with a forward step was used to build models with variables recording *P* values of ≤0.05 maintained.

Both FEC categorisation (positive vs. negative) and actual FEC were used as the categorical and continuous dependent variable for logistic and linear regression, respectively.

### Specific identification of rumen fluke eggs

Molecular analysis was completed on 54 samples from 14 individual farms belonging to counties Clare, Cork, Galway, Mayo, Roscommon and Laois, which recorded the highest FECs over the course of the study. Firstly, eggs were concentrated using the sedimentation technique described in [21]. Eggs were then washed in distilled water and collected individually using a modified fine-tip Pasteur pipette under stereomicroscope. DNA was extracted from eggs using the QIAamp® DNA stool mini kit (Qiagen, Hilden, Germany), at an increased lysis temperature of 95 °C for 5 min according to manufacturer’s instructions. To obtain the maximum DNA yield, two protocols were tested; (i) direct extraction of DNA from the isolated eggs; and, (ii) extraction following three freeze-thaw cycles at −80 °C for 30 min followed by boiling at 100 °C for 10 min. The concentration of DNA extracted using both methods was measured spectrophotometrically at 260 nm. Extracted DNA samples were stored at −80 °C until further analysis.

The ITS-2 region of ribosomal DNA was selected to allow effective discrimination between species [18]). ITS-2 from each of the samples was amplified by PCR using forward primer 5- TGCATACTGCTTTGAACATCG-3 and reverse primer 5-GTTCAGCGGGTATTCACGTC-3 [23]. Amplification was carried out in a 50 μl reaction volume which contained 5 μl buffer (10X), 1.5 μl 50 mM MgCl_2_, 1 μl of dNTP 10 mM, 1.5 U Taq DNA Polymerase (Invitrogen), and 1.25 μl of each primer 10 μM, and 5 μl of extracted DNA. Additionally, bovine serum albumin (BSA) was added to the PCR mixture to a final concentration of 0.2 μg/μl to facilitate amplification in presence of inhibitors. DNA of *C. daubnyei* (adult fluke) was added as positive control, and nuclease-free water included as negative control in all reactions. DNA samples from liver fluke were used to assess the specificity of the technique as well as extracted DNA from 200 mg of faeces from uninfected animals.

The reaction was completed in an Applied Biosystems 2700 thermocycler. The amplification parameters consisted of initial denaturation at 94 °C for 3 min, followed by 40 denaturation cycles (94 °C, 45 s), annealing (61 °C, 30 s) and extension (72 °C, 90 s), with a final extension phase at 72 °C for 10 min. The PCR products were analysed after electrophoretic separation in 1.5 % agarose gel and photographed using the Alphamanager 3400 (Alpha innotech) image capturer. The PCR products were purified using the Qiaquick Gel extraction kit and sequenced in forward and reverse directions by the Sanger (Beckman Genomics) method using the primers outlined earlier. The sequences obtained were edited using Editseq, and assembled using SeqMan tool (DNASnastar Inc., Madison, WI, USA) software to obtain a consensus sequence. Sequences were subsequently compared with those available in Genbank™, using the on-line **Standard Nucleotide** BLAST and the nucleotide collection (nr/nt) web interface provided by NCBI for specific identification [24]. The sequences were then aligned using MegAlign (DNASnastar Inc., Madison, WI, USA) software following the ClustalW (DNA Star) method.

## Results

### Descriptive analysis

A total of 304 flocks were recruited to the study while ensuring a representative geographical spread (Fig. 1). This yielded a sufficient sample size to achieve a 95 % confidence level and precision of 5 %, for a national sheep population of approximately 34,500 flocks with an expected national prevalence of 70 %. Of the 304 participating farmers that submitted samples over the course of the study, 253 (83 %) completed the management questionnaire.

The predominant breed on study farms was Suffolk and Suffolk crosses (40.3 %), with Texel and Texel crosses (23.3 %) the second most common. The remainder were a mixture of Charolais, Cheviot, Leicester, and Blackface Mountain. Median flock size across the study was 145 ewes (range 14 and 850). The majority of farmers (75.3 %) operated mixed enterprises (i.e. an additional livestock species present on the farm) and of these 68.6 % had beef cattle. With regard to grazing practices, over 187 flocks (74.5 %) use the same paddocks to graze all livestock together which, in the majority of cases, was lowland pasture (67.7 %). Approximately 50 % of participants operated mid-season lambing operations (March and April).

In relation to dosing practices, 190 farmers (75.0 %) stated that they dosed against rumen fluke once or twice over the autumn/winter period. The productused was oxyclozanide (*n* = 129; 50.9 %). A total of 61 farmers used a product not effective against rumen fluke, e.g. albendazole. Almost half of farmers that submitted a questionnaire (*n* = 123) reported not having had any clinical case of rumen fluke in the last five years. Only 10.3 % (*n* = 26) reported morbidity or mortality in sheep due to rumen fluke. Just over 40 % of farmers stated that they didn’t know if clinical cases of paramphisotomosis had ever occurred on their farms (*n* = 104). The distribution of study flocks across region, flock size, lambing season, and type of enterprise is included in Table 1.

**Table 1:** Distribution of study flocks across region, flock size, lambing-season and additional management factors

Region	Flock size			Mixed enterprise		Lambing period		Type of grazing		Organic farm	
	1–50	51–200	>200	Yes	No	Mid-season	Other season	Lowland	Mountain	Yes	No
High sheep density (n = 173)	*n* = 36	*n* = 101	*n* = 29	*n* = 98	*n* = 39	*n* = 88	*n* = 51	*n* = 116	*n* = 23	*n* = 14	*n* = 123
Low density (*n* = 131)	*n* = 19	*n* = 69	*n* = 39	*n* = 88	*n* = 22	*n* = 54	*n* = 55	*n* = 100	*n* = 10	*n* = 11	*n* = 98

### Prevalence

**Fig. 2:**
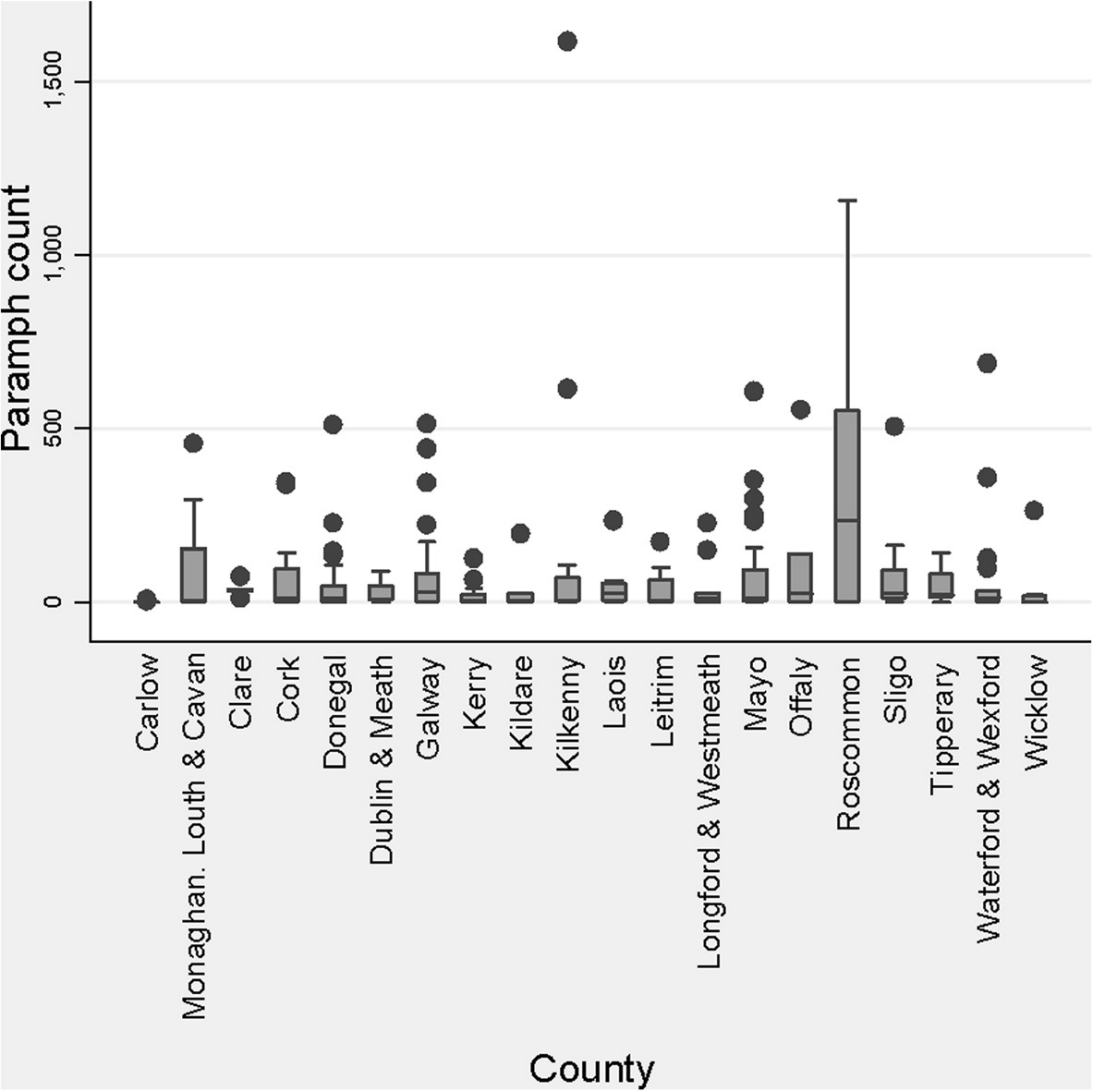
Distribution of mean epg of rumen fluke in each county

The apparent prevalence (Ap) and estimated true prevalence (Tp) of rumen fluke positive flocks was 77.3 (*n* = 235) and 85.7 %, respectively assuming a test sensitivity of 90 and specificity of 99.9 %. The prevalence across different counties ranged from 18.0 % in county Carlow to 100 % in counties Clare and Waterford. The Ap, Tp and number of farms recording FECs in low, medium and high categories is included in Table 2. The average parasite burden in positive flocks was 10.1 epg (range 0.1 epg to 161.6 epg). The farm recording the highest parasite burden of 161.6 epg was in Co. Kilkenny. The distribution of mean epg in each county is shown in Fig. 2. The level of infection in 85.5 of the flocks was low (<20 epg) and only 5.5 % recorded FECs of >50 epg. The Ap of rumen fluke in region 1 (high sheep density) was 87.7 % with a tendency to be higher than region 2 (low sheep density) where an Ap of 80.4 % was recorded (*P* = 0.083).

**Table 2:** Apparent (Ap) and true prevalence (Tp) of rumen fluke per region and number of farms recording FECs in low, medium and high categories

Region	Ap prevalence	True prevalence	Positive			Negative
			Low	Medium	High	
Region 1 High sheep densitiy	87,7	79	116	15	9	33
Region 2 Low sheep density	80,4	72,5	85	6	4	36

### Regression analysis

The final logistic regression model included the type of pasture and region as independent variables. The variables included in the final linear regression model were region, having different species mixed in the same grazing areas, and predominant sheep breed.

Results of the final logistic regression model are included in Table 3 and highlight a significant association between positive herd status and type of pasture grazed. Flocks grazing mainly lowland pastures were more than twice as likely to be infected compared with flocks grazing mountainous areas. A tendency existed for positive flocks to be located in more sheep dense regions than less dense regions (OR = 1.77). Outputs from linear regression highlighted a number of additional associations between actual FECs on each farm and independent variables such as breed, region and having different species mixed in the same grazing areas. Flocks in which Suffolk were the predominant breed recorded significantly higher epg than all other breed categories examined (Table 4).

**Table 3:** Logistic regression results of association between rumen fluke infection (positive vs. negative) as dependent variable and independent variables

Dependent variable	Independent variable	Odds Ratio	Confidence Interval (95 %)	*P* value	Variables in final model
Rumen fluke infection POSITIVE vs. NEGATIVE	Lowland pasture vs. mountain	2.35	1.06 5.21	0.036	Type of pasture Region *P* = 0.0013
	High density region vs. low density region	1.77	0.97 3.23	0.064

**Table 4:** Linear regression results of association between FEC as dependent variable and independent variables

Dependent variable	Independent variable	Coefficient	Confidence Interval (95 %)	*P* value	Variables in final model
Eggs per gram of faeces	Predominant breed Texal vs. Suffolk Mountain vs. Suffolk Other vs. Suffolk	−63.87	−131.74, −17.61	0.008	Predominant breed Region Sharing paddocks with other
		−59	−110.73, −7.26	0.026	
		−67.91	−120.30, −15.51	0.011	
	Region High density vs. low density	37.99	0.08, 75.90	0.049	
	Mixing livestock shared grazing vs. different paddocks	49.06	6.78, 91.34	0.023	

### Specific identification of eggs

The DNA extraction protocol from parasite eggs involving three cycles of freeze-thaw prior to isolation of DNA was found to yield optimal results, as no DNA was detected after PCR amplification without these cycles. The average concentration of DNA from one individual egg was 3.5 ng/μl (range 1.1 to 12.3 ng/μl). The PCR technique used allowed specific identification of eggs individually isolated from faecal samples. No cross reaction was detected with liver fluke DNA, nor uninfected samples. Amplified products were approximately 400 bp in size.

**Fig. 3:**
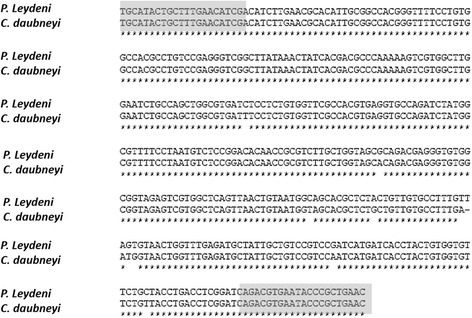
Alignment of ITS-2 sequences from both rumen fluke species obtained in the study (*C. daubneyi* and *P. leydeni*). The primers are highlighted in grey. ‘*’ indicates identical residues

Of the 54 individual eggs randomly selected from 14 farms, all sequences contained the complete ITS-2 region plus flanking 5.8S rRNA and 28S rRNA partial sequences. The majority of the samples sequenced (*n* = 52) were identified as *C. daubneyi*, and all 52 were identical. The sequences showed 100 % homology with the ITS-2 region of *C. daubneyi* (GenBank accession no. KP201674.1). The remaining two samples were identified as *P. leydeyni*. The pair of primers used produced a fragment of 402 bp in these samples, which showed 100 % homology to previously published sequences of the ITS-2 region of *P. leydeni* from northern Uruguay (GenBank accession no. KJ995529.1, gb|KJ995526.1, KJ995527.1) and Argentina (GenBank accession no. HM209064.1). The alignment of ITS-2 sequences from both species (*C. daubneyi* and *P. leydeni*) is shown in Fig. 3. No intraspecific variation was detected in any of the sequences obtained.

Both samples identified as *P. leydeni* were collected from a single farm in Co. Galway. This flock contained 66 sheep, and was a mixed enterprise with beef cattle grazing the same pastures as the sheep flock. All pastures were lowland and closantel was the active ingredient used for autumn and winter dosing. This farm also recorded a high infection level (34.3 epg) relative to the study farm population. No clinical cases of rumen fluke had been diagnosed on this farm within the previous five years, and the farm owner did not consider rumen fluke a health issue on his farm.

## Discussion

The recent emergence of paramphistomosis across Europe has resulted in a renewed interest in the study of this ruminant disease [25]. In this context, the national prevalence of rumen fluke in Irish sheep flocks and the associations with different risk factors has been investigated, along with the specific identification of eggs isolated from faecal samples.

The results obtained in this study revealed a true national prevalence of 85.7 %. As the bibliography related to herd-level prevalence is very rare, we have compared our results wth those obtained for individual animals in other countries. These results show that the prevalence in Ireland is considerably higher than those reported in other European countries. Other authors [25] reported a 22 % of prevalence in a field survey in Belgium; similarly in Spain a prevalence between 23.2 and 13.9 % was found in Galicia [14]; with comparable data recorded in France (20) and Italy (17 %) [26].

This study also highlights a higher prevalence in 2014 than that previously reported in Ireland [15, 16]. Between 2010 and 2013 [16], observed an increase in rumen fluke prevalence from 12 % to 22 % in sheep based on faecal examination. The large discrepancy between these data and the Ap reported in the current study (Ap 77.3 %) in 2014 is not likely reflective of a dramatic increase in the number of rumen fluke positive flocks in Ireland. It seems more plausible to attribute the differences in prevalence recorded to study design. In this previous study [16] a passive surveillance (i.e. voluntary submission of samples to regional veterinary laboratories for faecal screening) was used as a means of collecting prevalence data. Farmers submitting samples to veterinary laboratories are more likely to be proactive regarding parasite control on their farms, potentially introducing bias to that study population, thereby leading to an underestimation in prevalence. The current study aimed to reduce selection bias when recruiting farmers by targeting a large and nationally representative population of farmers. We would suggest, therefore, that the prevalence data presented here is more reflective of the national situation in Ireland compared to previous reports. The current study also serves to highlight the potential issues with using a convenient sample for determining prevalence as opposed to a targeted national study [27].

As many parasitic infestations are dependent on climatic conditions, it is important to examine weather conditions over time to highlight any potential contribution that changing weather could have on parasite prevalence [28].

Regardless of the underlying reasons for discrepancies across studies, it is important to examine why such a high prevalence of positive flocks was detected in the current study. A lack of implementation of an effective control programme would certainly appear to be a contributory cause. Even though most surveyed farmers dosed against rumen fluke, the regimens employed were markedly unsatisfactory. In the majority of cases, an anthelmintic active against rumen fluke was only administered once during the autumn/winter period. This is likely insufficient to reduce pasture contamination by viable eggs [29, 30]. Considering the complexity and length of the rumen fluke life cycle, coupled to the pasture-based management used in Ireland, a scientifically-based strategic dosing programme based on current epidemiological data ought to be evaluated. The over-reliance on oxyclozanide also requires a level of monitoring to ensure the development of resistance is not promoted [31].

Additional risk factors which were significantly associated with rumen fluke status included type of pasture grazed and breed. Similar to the relationship highlighted by other authors [14, 26] in Northern Spain, flocks predominantly grazing lowland pastures were more than twice as likely to be positive compared to those grazing mountain pastures. This is also consistent with the general epidemiology knowledge about pasture transmitted parasites, such as liver fluke. Additionally, a role for cows in acting as important reservoir of paramphistomosis infection has been previously proposed by [14]. Rumen fluke have been reported in Irish cattle [16], and it is not surprising, therefore, that study flocks which grazed different livestock species together were more heavily infected than those operating segregated grazing. The risk factors outlined in the current study can be used by veterinarians and farmers to design more appropriate dosing and grazing strategies to assist in the control of rumen fluke.

A renewed interest in the selection of animals with enhanced resistance to parasitic diseases has risen in the last decade [32] and selection for parasite resistance has shown to be effective in Australia and New Zealand [33]. In this context, an interesting and novel relationship between Suffolk breed and higher rumen fluke FEC was recorded in this study. Previous studies relating to parasite resistance in sheep have focused on gastrointestinal nematodes and have established, both in Ireland [34, 35] and internationally [36] that Suffolks are less resistant to nematode infections than other breeds. This study provides evidence that the Suffolk breed may also be more susceptible to rumen fluke parasitism. Given the considerable number of Suffolk sheep in Ireland^3^, a more comprehensive investigation into the current finding is warranted with investigations also expanded to include liver fluke. With the current emphasis on sustainable farming across global livestock enterprises [37], any opportunity to breed more disease resistant and resilient animals should be thoroughly investigated.

Although identification of parasites at species level has proven to be crucial for epidemiological studies, the majority of reports of paramphistomosis do not define the species of rumen fluke present, as eggs from different species cannot be differentiated using morphoanatomical identification. Identification of eggs using molecular techniques, therefore, proves beneficial in this regard. In the current study, a PCR technique described previously by [23] was carried out to identify individual eggs isolated from faecal samples. The most noteworthy finding was the identification *P. leydeni* infecting sheep flocks in Ireland for the first time. This unexpected result contrasts with previous reports where *C. daubneyi* was considered the only species affecting livestock, both in Western Europe [12–14, 38], and Ireland [15, 16]. The majority of previous studies carried out in Ireland completed species identification on adult parasites collected at abattoirs. Zintl and coworkers [15] collected 70 adult flukes from two cattle herds and Toolan [16] examined adult specimens from 25 cattle and 11 sheep. *C. daubneyi* was the only species identified. It should be noted that no intraspecific differences were detected in either rumen fluke species, which agrees with results obtained previously by other authors [39, 40], who sequenced samples of *P. leydeni* adults, and [18] who characterised samples of *C. daubneyi* from different hosts.

Based on identification of *P. leydeni* in the current study, more comprehensive investigations are required into the range of paramphistomes present in domestic livestock. *P. leydeni* is a common parasite in Irish deer [41] whichmay be acting as potential wildlife reservoir for livestock infection. Alternatively, identification of *P. leydeni* in this flock may be an incidental finding specific to this flock alone. Whether the presence of *P. leydeni* contributes to morbidity in sheep hosts in Ireland is unknown, especially as the farm on which this species was identified did not report an issue with rumen fluke. Based on our literature search, this is the first report of *P. leydeni* infecting livestock in Europe although the consequences of this finding remain unclear. However, in Argentina it might represent an issue in some regions, and an increase in the spread of *P. leydeni* over the years in Argentina has been observed [39].

Despite the increasing prevalence of rumen fluke in temperate regions, its impact on flock performance and associated economic losses remains uncertain. The isolation of individual eggs method coupled with PCR technique described here, could facilitate the study of the production outcomes associated with differing species of rumen fluke. Identification of paramphistomes based on collection of adult fluke in abattoirs is troublesome in this regard, as an individual animal may undergo a number of farm movements prior to slaughter making identification of the most likely source of infection problematic. Identification of paramphistome species using faecal egg isolation on the farm of origin, as described in the current study, avoids the necessity to trace animal movements prior to slaughter. This facilitates collection of animal and herd specific production data and investigation of the impact varying species of rumen fluke may have on livestock performance.

Although a positive correlation between rumen fluke burden and faecal consistency has previously been reported [25], few studies of this kind exist, and the pathogenic importance of rumen fluke is disputed [42]. One of the main setbacks to carry out these studies is the lack of reliable diagnostics tests to detect immature rumen flukes. This has already been stressed by other authors [16], who emphasised that the mortality attributed to rumen fluke is not correlated with prevalence of infection, measured by faecal examination. In that respect, the fact that 40 % of farmers stated a lack of knowledge about the occurrence of clinical cases of paramphistomosis is a reflection of the absence of such diagnostic tests. The requirement for a rapid, immunological test to detect antigen from immature rumen fluke should be urgently addressed given the prevalence of adult rumen fluke highlighted in our study.

## Conclusions

This study has identified an exceptionally high prevalence of rumen fluke in Irish sheep flocks and several risk factors contributing to infection. The identification of *P. leydeni* infection in sheep in Ireland for the first time is a novel finding although the implications of this outcome remain still to be elucidated. The increase of anthelmintic resistance worldwide has emphasized the importance of management strategies in parasite control, in that regard, the novel association between Suffolk breed and higher FEC found in this study could provide with a new line of research on breeding rumen fluke resistant or resilient sheep.

## Abbreviations

DNA, deoxyribonucleic acid, epg, eggs per gram of faeces, FEC, faecal egg count, ITS 2, Internal transcribed spacer 2, OR, odds ratio, PCR, Polymerase chain reaction, rRNA, ribosomal ribonucleic acid, TP, true prevalence, AP, apparent prevalence

## Footnotes

1 https://www.agriculture.gov.ie/media/migration/agri-foodindustry/foodharvest2020/2020FoodHarvestEng240810.pdf

2 www.surveymonkey.com

3 http://www.fao.org/ag/agp/agpc/doc/counprof/ireland/ireland.htm
